# *T*_2_-weighted MRI detects presymptomatic pathology in the SOD1 mouse model of ALS

**DOI:** 10.1038/jcbfm.2014.19

**Published:** 2014-02-05

**Authors:** Matthew C Evans, Sébastien Serres, Alexandre A Khrapitchev, Helen B Stolp, Daniel C Anthony, Kevin Talbot, Martin R Turner, Nicola R Sibson

**Affiliations:** 1 Nuffield Department of Clinical Neurosciences, University of Oxford, Oxford, UK; 2 CR-UK/MRC Gray Institute for Radiation Oncology and Biology, Department of Oncology, University of Oxford, Oxford, UK; 3 Department of Physiology, Anatomy and Genetics, University of Oxford, Oxford, UK; 4 Department of Pharmacology, University of Oxford, Oxford, UK

**Keywords:** amyotrophic lateral sclerosis, apparent diffusion coefficient, blood–brain barrier, biomarker, magnetic resonance imaging, magnetization transfer ratio

## Abstract

Neuroinflammation has been identified as a potential therapeutic target in amyotrophic lateral sclerosis (ALS), but relevant biomarkers are needed. The superoxide dismutase (*SOD1)*^*G93A*^ transgenic mouse model of ALS offers a unique opportunity to study and potentially manipulate presymptomatic pathology. While *T*_2_-weighted magnetic resonance imaging (MRI) has been shown to be sensitive to pathologic changes at symptom onset, no earlier biomarkers were previously identified and the underlying histopathologic correlates remain uncertain. To address these issues, we used a multimodal MRI approach targeting structural (*T*_2_, *T*_1_, apparent diffusion coefficient (ADC), magnetization transfer ratio (MTR)), vascular (gadolinium diethylene triamine pentaacetic acid), and endothelial (vascular cell adhesion molecule–microparticles of iron oxide) changes, together with histopathologic analysis from presymptomatic to symptomatic stages of disease. Presymptomatic changes in brainstem nuclei were evident on *T*_2_-weighted images from as early as 60 days (*P*<0.05). Histologic indices of vacuolation, astro- and microglial activation all correlated with *T*_2_-weighted changes. Significant reductions in ADC (*P*<0.01) and MTR (*P*<0.05) were found at 120 days in the same brainstem nuclei. No changes in *T*_1_ relaxation, vascular permeability, or endothelial activation were found at any stage of disease. These findings suggest that *T*_2_-weighted MRI offers the strongest biomarker potential in this model, and that MRI has unique potential for noninvasive and longitudinal assessment of presymptomatically applied therapeutic and neuroprotective agents.

## Introduction

Amyotrophic lateral sclerosis (ALS) is an adult-onset neurodegenerative disorder characterized pathologically by a progressive loss of upper motor neurons of the corticospinal tract and lower motor neurons of brainstem nuclei and ventral roots of the spinal cord. There is currently no treatment to reverse progression, and the median survival from symptom onset is 2 to 4 years.^1^

The best characterized and most consistent model of ALS is the transgenic rodent overexpressing mutant forms of the human Cu/Zn superoxide dismutase (*SOD1*) gene, in particular *SOD1*^*G93A*^.^2^ Superoxide dismutase mice have a predictable disease progression and pathology, and uniquely recapitulate some of the core clinical features of the human disorder.^3^ The *SOD1*^*G93A*^ rat, being larger, might offer advantages for magnetic resonance imaging (MRI) but has been less well characterized.

The capacity of the *SOD1*^*G93A*^ mouse model to successfully deliver disease-modifying agents that have positive effects on symptomatic human ALS has been disappointing, but its unique window on presymptomatic pathologic events has the potential to inform future neuroprotective therapeutic trials. The need for an optimized and standardized framework for such animal studies is now recognized.^4^

In addition to survival, slowing of the decline in motor function is currently the primary biomarker in such trials. The rotarod apparatus is typically used to measure symptom onset and progression,^5^ and is traditionally an important marker when evaluating the effectiveness of drugs. However, symptoms only become apparent with this approach when the underlying pathology is significantly advanced, and behavioral tests are necessarily only sensitive to function of specific neuronal populations. Magnetic resonance imaging in contrast offers unique potential to noninvasively provide information about underlying pathologic changes that occur in all regions of the central nervous system before, during, and after the onset of symptoms.^6^

While it is established that *T*_2_ MRI is sensitive to tissue changes around the time of symptom onset in *SOD1* mice,^7, 8, 9^ it is not yet clear which aspects of pathology are measured by this technique, although vacuolation is a hypothesis.^8^ Moreover, MRI indices of presymptomatic pathology have yet to be identified, and work with other MRI approaches is relatively scarce and largely restricted to diffusion-weighted MRI.^7, 10^ Nonimaging studies have shown vascular alterations at the blood–brain barrier (BBB) in *SOD1* mouse models, including increased permeability and vascular activation.^11, 12, 13^ Similarly, MRI studies in the *SOD1*^G93A^ rat model using the contrast agent gadolinium diethylene triamine pentaacetic acid (Gd-DTPA) have shown increased BBB permeability in the midbrain/interbrain region.^14, 15^ At the same time, cellular adhesion molecules such as vascular cell adhesion molecule 1 (VCAM-1) that are upregulated during endothelial activation can be tracked *in vivo* using MRI by conjugation of appropriate antibodies to microparticles of iron oxide (MPIO).^16, 17^ However, neither of these contrast agent approaches have yet been applied in transgenic mouse models of ALS.

The aim of this study was to use a multimodal MRI approach, including *T*_1_ (with and without Gd-DTPA), *T*_2_, *T*_2_* (with VCAM-MPIO), apparent diffusion coefficient (ADC), and magnetization transfer ratio (MTR), together with histologic assessment of pathology, to determine their potential as early and sensitive biomarkers for ALS disease progression, and to characterize the histologic substrate of the magnetic resonance changes observed.

## Materials and methods

### Mice

Transgenic mice carrying a mutation in the human *SOD1* gene derived from the B6SJL-TgN (*SOD1*^G93A^) model from the Jackson Laboratories (Bar Harbor, ME, USA) were used. The line was maintained by mating transgenic male mice with wild-type (WT) B6SJL mice, acquired from Harlan Laboratories (Bicester, UK). Mice were housed in individually ventilated cages, and given food and water *ad libitum*. All animal experiments were approved by the University of Oxford local animal ethical committee, and performed according to terms of a license granted by the UK Home Office (License number 30/2533), adhering to the Animals (Scientific Procedures) Act 1986. Female SOD1^G93A+/−^ mice were used as the experimental group and female WT (SOD1^G93A−/−^) littermates were used as a control group. This allowed all male SOD1^G93A+/−^ to be used to maintain the colony.

### Behavioral Assessments

Mice were tested on the rotarod (Ugo Basile, Comerio, Italy) at 40, 60, 80, 100, and 120 days, measuring time taken to fall off the apparatus. A larger sample (*N*=10) in each group was used for behavioral measurements (including the mice used for the MRI study), to increase the study power. Mice were also weighed just before each behavioral session. The combination of these two methods allowed assessment of symptom onset.

### Magnetic Resonance Imaging

Monoclonal antibodies to VCAM-1 (clone M/K2; Cambridge Bioscience, Cambridge, UK) were conjugated to myOne tosylactivated MPIO (1 *μ*m diameter; Invitrogen, Carlsbad, CA, USA), as described previously.^16^

Female *SOD1*^*G93A*^ and female WT littermates in each group were scanned once only, and euthanized immediately after the MRI to allow histologic analysis. Magnetic resonance imaging was performed using a 7-T superconducting magnet driven by a Varian DirectDrive spectrometer (Varian Inc., subsidiary of Agilent Technologies, Santa Clara, CA, USA) at 40, 60, 80, 100, and 120 days (*N*=4 in each group, at each time point). Mice were anesthetized by inhalation of 1.5% isoflurane with 30% O_2_ and 70% N_2_, and placed in a quadrature radio frequency coil with tooth bar and head restraint to reduce motion artifact. Respiration was monitored throughout and body temperature was maintained at 37°C.

For detection of VCAM-MPIO, a *T*_2_*-weighted 3D gradient echo data set was acquired as follows: flip angle=27° repetition time (TR)=65 ms; echo time (TE)=7.5 ms; field of view=22.5 mm × 22.5 mm × 11.25 mm; matrix size=256 × 192 × 96; averages=2; total acquisition time ca. 40 minutes. The midpoint of acquisition was 1.5 hours after MPIO injection (4 mg Fe/kg body weight in 100 *μ*L saline, intravenously). Data were zero-filled to 256 × 256 × 128 and reconstructed off-line, giving a final isotropic resolution of 88 *μ*m.

Following, a fast spin-echo multislice sequence (*T*_2_-weighted) was acquired spanning the brain and the brainstem: TR=3,000 ms; TE=61.9 ms; field of view=25 × 25 mm; matrix size=256 × 256; ns=13; averages=2; slice thickness=1 mm; acquisition time=3 minutes 12 seconds.

Subsequently, *T*_1_-weighted images were acquired using a spin-echo sequence (TR=500 ms; TE=20 ms; field of view=25 mm × 25 mm; matrix size=256 × 256; ns=11; averages=2; slice thickness=1 mm; acquisition time=4 minutes 16 seconds) both pre-Gd-DTPA and 5 minutes post-Gd-DTPA (Omniscan; GE Healthcare, Chalfont, UK) injection (30 *μ*L; intravenously) to identify BBB permeability, as described previously.^18, 19^ Soft tissues in the head were examined to confirm proper delivery of Gd-DTPA, which extravasates rapidly into these tissues.

In a separate group of *SOD1*^*G93A*^ and WT mice (*N*=5 per group), *T*_2_-weighted (as above), diffusion-weighted, and magnetization transfer data were acquired at 120 days. Diffusion-weighted images were acquired using a navigated pulsed-gradient spin-echo sequence (TR=2.0 seconds; TE=35.1 ms; matrix size=128 × 128; average=1; slice thickness=1 mm; ns=11; total acquisition time=12 minutes 48 seconds each), with diffusion-weighting *b* values of 125, 500, and 1,000 s/mm^2^, a diffusion gradient duration of 12.5 seconds, gradient separation of 17.5 ms and gradient amplitude of 8.19 G/cm. Diffusion gradients were applied separately along three orthogonal axes and ADC ‘trace' maps were calculated.

To obtain MTR maps, spin-echo images were acquired (TR=4.5 seconds; TE=13.8 ms; hard magnetization transfer pulse length=384 ms; offset frequency=−1,500 Hz; field of view=25 × 25 mm; matrix size=128 × 128; ns=11; average=1; slice thickness=1 mm) with and without the magnetization transfer saturation pulse (total acquisition time=9 minutes 35 seconds each scan).

### Magnetic Resonance Imaging Analysis

All acquisition, processing, and calculations were performed in VNMRJ (Agilent Technologies) unless otherwise specified. For analysis of the 3D *T*_2_* data, whole brain, cortex, and brainstem regions were segmented using ITK snap (http://www.itksnap.org/pmwiki/pmwiki.php). Within each of these regions, the number of hypointensities was calculated using Matlab (Mathworks Inc., Natick, MA, USA). Hypointensities were calculated as those voxels that were <65% of the mean signal intensity of all voxels in the tissue, and a cutoff of 20 contiguous voxels was used to omit larger structures (e.g., sinuses). By dividing the number of hypointensities by the number of voxels in the mask, the percentage of hypointense voxels per brain was calculated.

For *T*_1_ data sets, the pre-Gd-DTPA image was subtracted from the post-Gd-DTPA image, and expressed relative to the pre-Gd-DTPA image. For *T*_1_- and *T*_2_-weighted images, and ADC and MTR maps, circular regions of interest (ROIs) were placed over the three motor nuclei (MN V, VII, and XII) in each mouse in native space, as well as a control region on the same slices as the nuclei (within the brainstem, but outside the nuclei of interest), and intensities were measured as (ROI−control region)/control region. The ROI size was set to adequately cover the area of hyperintensity in 120-day *SOD1*^*G93A*^ mice, which encompasses the entire area of the motor nucleus. Diameters were trigeminal, 1.4 mm^2^; facial, 1.2 mm^2^; hypoglossal, 1.1 mm^2^.

### Intracerebral Injections

To obtain positive control tissue for immunohistochemistry and VCAM-1 contrast, mice were microinjected bilaterally with human recombinant interleukin-1*β* (IL-1*β*) (1 ng/*μ*L). Mice were anesthetized as described for MRI above, and cytokine was stereotactically microinjected over 5 minutes into the sensorimotor cortex (0.5 mm anterior and 1.5 mm lateral (left and right) to Bregma, at a depth of 0.75 mm from the cortical surface), using a finely drawn glass microcapillary (Drummand Scientific Company, Broomall, PA, USA). For brain trauma control, the same procedure was performed with a large bore (23 G) needle with no injection of cytokine. Mice were perfusion fixed (as below) for histology 4 hours after cytokine injection.

### Histology/Immunohistochemistry

Following MRI, mice were killed using pentobarbital (100 *μ*L) and transcardially perfused with heparinized saline followed by transcardial fixation with 100 mL PLP_light_ (75 mmol/L lysine HCl, 10 mmol/L disodium hydrogen orthophosphate, 10 mmol/L sodium periodate, 2% paraformaldehyde, 0.025% glutaraldehyde). Brains were removed and cut in half down the midline. One hemisphere was further fixed with 4% paraformaldehyde for 24 hours, and then dehydrated through increasing grades of ethanol (80%, 95%, and 100%), cleared in histoclear and embedded in paraffin wax. The second hemisphere was further fixed in PLP_light_ for 4 hours, and then placed in 30% sucrose for cryoprotection. Following cryoprotection, the tissue was frozen in TissueTek OCT mounting medium (Bayer Plc, Newbury, UK) using isopentane cooled on dry ice. Wax sections were cut at 10 *μ*m using a Reichart-Jung microtome (Heidelberg, Germany). Frozen sections were cut at 10 *μ*m using a Leica CM1850 cryostat (Leica Microsystems, Milton Keynes, UK).

Sections through the brainstem nuclei were stained with H&E to study vacuolation (wax sections). In addition, immunohistochemistry was performed using antibodies to glial fibrillary acidic protein (GFAP) (rabbit anti-GFAP; DAKO, Glostrup, Denmark) for activated astrocytes (wax sections), ionized calcium-binding adapter molecule 1 (Iba1) (goat anti-Iba1; Abcam, Cambridge, UK) for activated microglia (wax sections), and VCAM-1 (rat anti-VCAM-1; Cambridge Bioscience) for vascular activation (frozen sections). Wax sections were first rehydrated, and subsequently washed in phosphate-buffered saline (wax and frozen sections). Endogenous peroxidase activity was quenched using methanol with 0.3% H_2_O_2_ for 20 minutes, and Fc sites blocked using 10% serum (goat serum for GFAP and VCAM-1, horse serum for Iba-1) for 1 hour at room temperature. Primary antibody was diluted in phosphate-buffered saline (1:1,000 for GFAP, 1:500 for Iba-1, and 1:200 for VCAM-1), and incubated with the tissue overnight at 4°C. Appropriate secondary antibody (Vector Labs, Peterborough, UK) was added to the tissue 1:100 for 1 hour (goat anti-rabbit IgG for GFAP, horse anti-goat IgG for Iba-1, aand goat anti-rat IgG for VCAM-1), followed by Vector elite ABC reagent for 1 hour. All tissue of the same time point, stained with the same antibody, were processed at the same time to ensure consistency. Antibody binding was visualized using the 3,3′-diaminobenzidine (DAB) reaction, and counterstaining for immunohistochemistry was with hematoxylin alone.

### Analysis of Histology/Immunohistochemistry

For immunohistochemistry analysis, the three largest cross-sections of each nucleus were used for consistency (V, VII, and XII), using a × 10 objective lens. Nuclei were localized using the Paxinos mouse brain atlas as a reference.^20^ Images were then analyzed using the ImageJ software (National Institute of Health, Bethesda, MD, USA). Color deconvolution was used to split the brown DAB channel from the blue hematoxylin channel, and the mean optical density of the DAB stain was measured in a standard circle with a diameter of 500 units within ImageJ. Optical density (i.e., absorbance) is the log ratio of the amount of light being absorbed by the tissue, and the amount of light passing through. This increases with the amount of DAB deposition and, therefore, staining intensity. The optical density measure was then averaged across the three slices through the nucleus.

For the analysis of vacuolation using H&E-stained tissue, photographs were taken of the largest three cross-sections of the nuclei using a × 10 objective lens using the Paxinos atlas for reference.^20^ Analysis was also performed using ImageJ. The background was subtracted, and the image was binarized to give a value of 0 for normal tissue and 1 for pixels within vacuoles. An ROI was then drawn around the area of tissue affected by vacuolation, and the area and the average intensity were measured. From this, total vacuolation was calculated (area × average vacuolation) and used for analysis.

### Statistical Analysis

Sample sizes were chosen based on previous studies, and our experience with the techniques employed. ANOVA with *post hoc t*-tests (Hulm–Bonferroni) was used for comparisons between groups (SPSS, IBM, Armonk, NY, USA). Effect sizes were compared between MRI sequences using Cohen's *d,* calculated from the mean difference and the standard deviation. Pearson's *r* was calculated for the correlation of *T*_2_ hyperintensity in each brainstem nucleus with (1) quantification of vacuolation, (2) GFAP staining intensity, and (3) Iba1 staining intensity. To compare correlation coefficients, *r* was converted into *r*' using Fisher's transformation, and from this a z score was calculated for statistical analysis (Microsoft Excel).

## Results

### Behavioral Data

**Figure 1:**
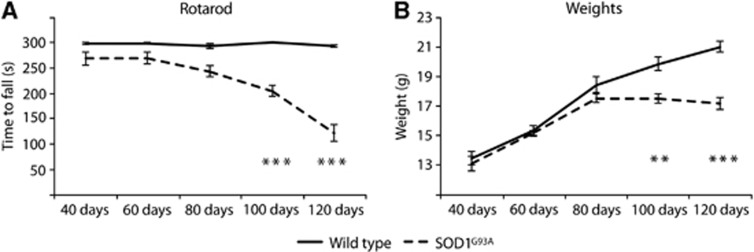
Rotarod scores and weights across lifespan for *SOD1*^*G93A*^ and wild-type (WT) mice. There was a gradual deterioration in motor score over time in *SOD1*^*G93A*^, but not in WT mice (**A**), with a significant reduction from baseline in motor behavior at 100 and 120 days. Weights show a stereotypical pattern (**B**), with WT mice increasing in body weight in a linear manner, but the *SOD1*^*G93A*^ group becoming significantly lighter at 100 and 120 days. **P<0.01; ***P<0.001. *N*=10 in each group. *SOD1*, superoxide dismutase.

In keeping with other studies, *SOD1*^G93A^ mice showed an age-dependent reduction in motor performance as measured by rotarod testing, with statistically significant separation from WT controls at all time points (*P*<0.05), and a decline in performance from baseline in *SOD1*^G93A^ mice at 100 and 120 days (*P*<0.001). No significant differences were found within the WT group over time. Weight data (Figure 1B) showed a similar pattern, with *SOD1*^*G93A*^ mice displaying a significant weight loss compared with WT littermates at 100 days (*P*<0.01) and 120 days (*P*<0.001). Therefore, using both these measures, symptom onset occurred between 80 and 100 days of age, consistent with previous reports of this model.^21^

### *T*_2_- and *T*_1_-Weighted Magnetic Resonance Imaging

**Figure 2:**
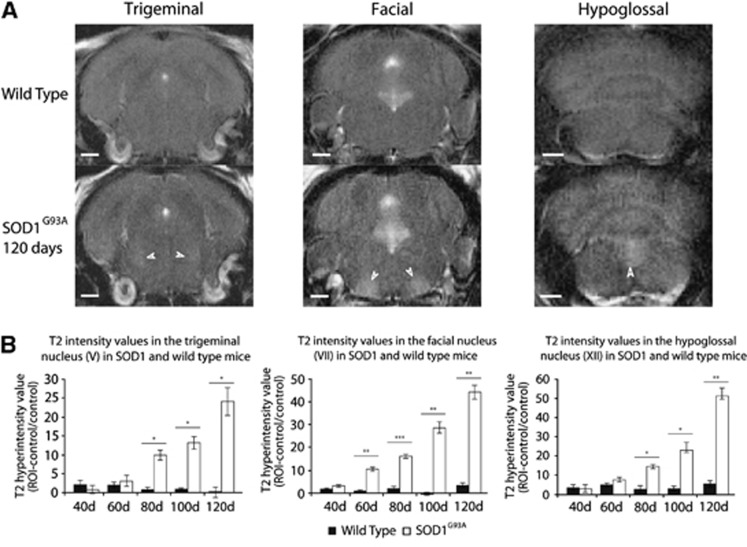
Comparison of *T*_2_ changes in motor nuclei V, VII, and XII in *SOD1*^*G93A*^ and wild-type (WT) mice. Representative *T*_2_ images of WT and *SOD1*^G93A^ mice at 120 days (**A**) in nuclei V (left panels), VII (middle panels), and XII (right panels) show a marked signal enhancement in *SOD1*^*G93A*^ mice, compared with surrounding brainstem. Quantification of signal change in the regions of interest (ROIs) compared with surrounding brainstem region (**B**) shows an early (60 days in nucleus VII) and progressive increase in signal in *SOD1*^*G93A*^ but not in WT mice. *P<0.05; **P<0.01; ***P<0.001. *N*=4 in each group. Scale bars represent 1 mm. *SOD1*, superoxide dismutase.

*T*_2_-weighted MRI revealed an early and progressive degenerative change in motor nuclei V (trigeminal), VII (facial), and XII (hypoglossal) compared with the control brainstem region (Figure 2). There was significantly more degenerative change as measured by *T*_2_ intensity, in *SOD1*^G93A^ compared with WT mice (V *P*<0.05; VII *P*<0.01; XII *P*<0.001), which increased with disease progression (V *P*<0.01; VII *P*<0.001; XII *P*<0.01). A significant difference was evident between *SOD1*^*G93A*^ and WT in all three nuclei at 80 days (V *P*<0.05; VII *P*<0.001; XII *P*<0.05), 100 days (V *P*<0.05; VII *P*<0.01; XII *P*<0.05), and 120 days (V *P*<0.05; VII *P*<0.01; XII *P*<0.01), while a difference in the facial nucleus (VII) was found at the earlier time point of 60 days (*P*<0.01). At all time points there was a greater effect size for the difference in T_2_ intensity between *SOD1*^*G93A*^ and WT mice in nucleus VII (Supplementary Table 1).

In comparison with the *T*_2_ signal change, no significant changes were seen between *SOD1*^*G93A*^ and WT mice using *T*_1_-weighted imaging at any time point. Representative images and quantification of *T*_1_ signal intensity are shown in Supplementary Figure 1.

### Comparison of *T*_2_, Apparent Diffusion Coefficient, and Magnetization Transfer Ratio at 120 Days

**Figure 3:**
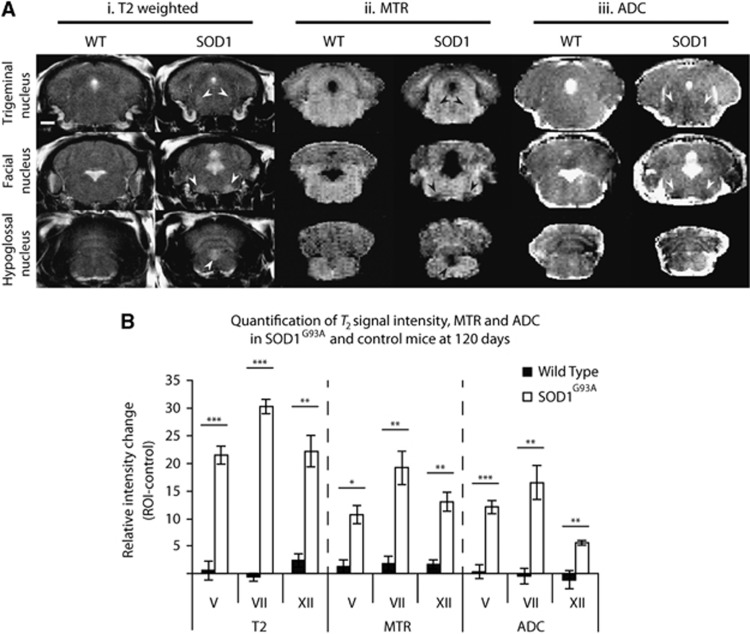
Comparison of *T*_2_, magnetization transfer ratio (MTR), and apparent diffusion coefficient (ADC) sequences in *SOD1*^*G93A*^ and control mice at 120 days. Representative images from *T*_2_ (i), MTR (ii), and ADC (iii) sequences (**A**) showed visible changes in *SOD1*^*G93A*^ mice for all nuclei (arrows) except for nucleus XII for ADC. Quantification of intensity changes in *T*_2_, MTR, and ADC sequences in *SOD1*^*G93A*^ and control mice (**B**) indicate that a significant intensity change occurred for all three nuclei in all sequences. Note that intensity change for ADC and MTR has been inverted for comparison with *T*_*2*_. *P<0.05; **P<0.01; ***P<0.001. *N*=5 in each group. Scale bar represents 1 mm, and applies to all images. *SOD1*, superoxide dismutase; ROI, region of interest.

A second cohort of animals were scanned with *T*_2_, MTR, and ADC sequences at 120 days. As seen in the cross-sectional experiment above, there was a significant increase in *T*_2_ signal intensity at 120 days in all three brainstem motor nuclei (*P*<0.001) compared with the control brainstem region (arrows, Figure 3Ai) in *SOD1*^*G93A*^ but not in WT mice. For the MTR data, a significant decrease in signal intensity was found in nuclei V (*P*<0.05), VII (*P*<0.01), and XII (*P*<0.01) compared with the control brainstem region in *SOD1*^*G93A*^ mice (arrows, Figure 3Aii). Similarly, a significant decrease in ADC was found in the *SOD1*^*G93A*^ group in nuclei V (*P*<0.001), VII (*P*<0.01), and XII (*P*<0.01). However, for the ADC data, while changes in nuclei V and VII were apparent by eye in the images (arrows, Figure 3Aiii) changes in nucleus XII were subtler owing to artifacts from the nearby ventricle.

Effect sizes were calculated for the difference between *SOD1*^*G93A*^ and WT mice in the three nuclei at 120 days (Supplementary Table 2). *T*_2_ changes were associated with a substantially greater effect size in all three nuclei, particularly the facial nucleus. The effect size of the MTR and ADC changes was comparable in the three nuclei, with a marginally greater effect size for MTR in nucleus XII, and for ADC in nucleus V.

### Histologic Analysis of Vacuolation

**Figure 4:**
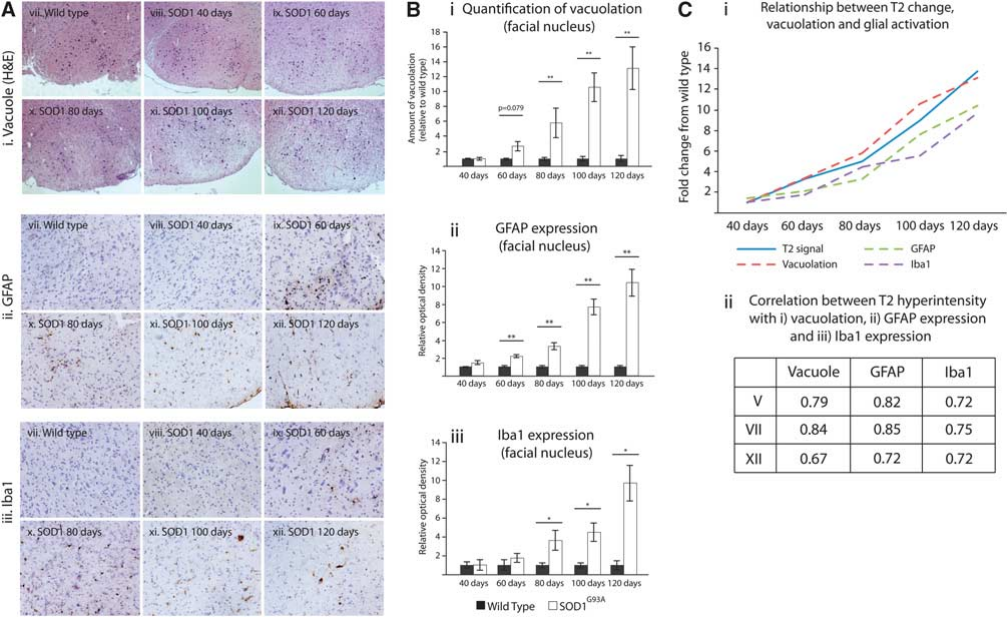
Relationship between *T*_2_ signal change with vacuolation, astrocyte activation (GFAP), and microglia activation (Iba1). Representative images are shown for vacuolation (H&E) (i), and GFAP (ii), and Iba1 (iii) immunoreactivity in wild type (WT) mice and *SOD1*^*G93A*^ throughout the disease in the facial nucleus (**A**). Hematoxylin counterstain was used for immunostaining. (**B**) Quantification of these disease features showing an increase in vacuolation (i), GFAP staining (ii), and Iba1 staining (iii) in the facial nucleus over from 60/80 days to 120 days. By plotting fold change in *T*_2_ hyperintensity, vacuolation, and GFAP/Iba1 immunoreactivity (**C**i), we can see a similar relationship between *T*_2_ intensity changes with all three variables (vacuolation, GFAP, and Iba1). In line with this, there is a high correlation coefficient (Pearson's *r*) for the relationship with *T*_2_ intensity change for vacuolation, GFAP immunoreactivity, and Iba1 immunoreactivity in all three cranial nuclei (V, VII, and XII) (**C**ii), with no significant difference between the correlation coefficients in each of these regions (*P*>0.05). *P<0.05; **P<0.01, GFAP, glial fibrillary acidic protein; Iba1, ionized calcium-binding adapter molecule 1; *SOD1*, superoxide dismutase.

Mirroring the *T*_2_ MRI data, there was a progressive development of tissue vacuoles over time (*P*<0.05), in *SOD1*^G93A^ but not in WT mice (*P*<0.05). In nucleus V (Supplementary Figure 2Ai–vi and Bi), a trend toward increased vacuolation could be seen as early as 60 days (*P*=0.061), while a significant increase in vacuolation was found at 80 (*P*<0.05), 100, and 120 days (*P*<0.01). In nucleus VII (Figures 4Ai and 4Bi; Supplementary Figure 2Avii–xii and Bii), vacuolation also appeared to be increased at 60 days (*P*=0.079), reaching significance from 80 days of age (*P*<0.01; 80, 100, and 120 days). In nucleus XII (Supplementary Figure 2Axiii–xviii and Biii), a trend toward increased vacuolation was again apparent, but not significant, at 60 days (*P*=0.087), and reached significance from 80 days (*P*<0.05; 80, 100, and 120 days).

### Astrocyte Glial Fibrillary Acidic Protein Immunohistochemistry

For all three nuclei, significantly stronger staining of GFAP was seen in *SOD1*^*G93A*^ compared with WT mice (*P*<0.01), which increased over time (*P*<0.05). Within nucleus V (Supplementary Figure 3Ai–vi and Ci), there was a trend toward increased GFAP immunoreactivity at 60 (*P*=0.059) and 80 days (*P*=0.084), which was significantly elevated at 100 days (*P*<0.01). In nucleus VII (Figures 4Aii and 4Bii; Supplementary Figure 3Avii–xii and Cii), GFAP immunoreactivity was significantly increased from 60 to 120 days of age in *SOD1*^*G93A*^ compared with WT mice (*P*<0.01). A significant increase in GFAP immunoreactivity in nucleus XII (Supplementary Figure 3Axiii–xviii and Ciii) was evident from 80 to 120 days (*P*<0.05).

### Microglial (Iba1) Immunohistochemistry

As was the case for GFAP, a significant increase in Iba1 staining was seen in all three nuclei for *SOD1*^*G93A*^ compared with WT mice (*P*<0.05), which increased over time (*P*<0.05). For nucleus V (Supplementary Figure 4Ai–vi and Ci), there was a trend to increased Iba1 expression in *SOD1*^*G93A*^ mice at 80 days (*P*=0.071), which was significantly increased at 100 and 120 days (*P*<0.01). In both nuclei VII and XII (Figures 4Aiii and 4Biii; Supplementary Figure 4Avii–xii, Cii, Axiii–xviii and Ciii, respectively), Iba1 staining was significantly greater from 80 to 120 days of age (*P*<0.05) in *SOD1*^*G93A*^ mice compared with WT controls.

### Correlation of Magnetic Resonance Imaging Changes with Histologic Measures of Vacuolation and Glial Immunoreactivity

Vacuolation became evident from around the same time as the increased *T*_2_ signal, and increased progressively throughout the disease in a way that was comparable to *T*_2_ signal change (Figures 4Ai, 4Bi, and 4Ci). However, in addition to this, activation of astrocytes (Figures 4 Aii, 4Bii, and 4Ci) and microglia (Figures 4Aiii, 4Biii, and 4Ci), as shown by an increase in expression of GFAP and Iba1, respectively, also started from approximately the time of signal change on *T*_2_ MRI, and increased as the disease developed. These data are shown for the facial nucleus, but are also consistent with data from the trigeminal and hypoglossal nuclei. Correlational analysis (Figure 4Cii) showed a very high correlation between all three variables (vacuolation, GFAP, and Iba1) and *T*_2_ intensity change, but there was no significant difference between Pearson's *r* for any of the correlations in any of the regions, showing a similar strength of association.

### Magnetic Resonance Imaging indices of Blood–Brain Barrier Permeability and Vascular Activation

**Figure 5:**
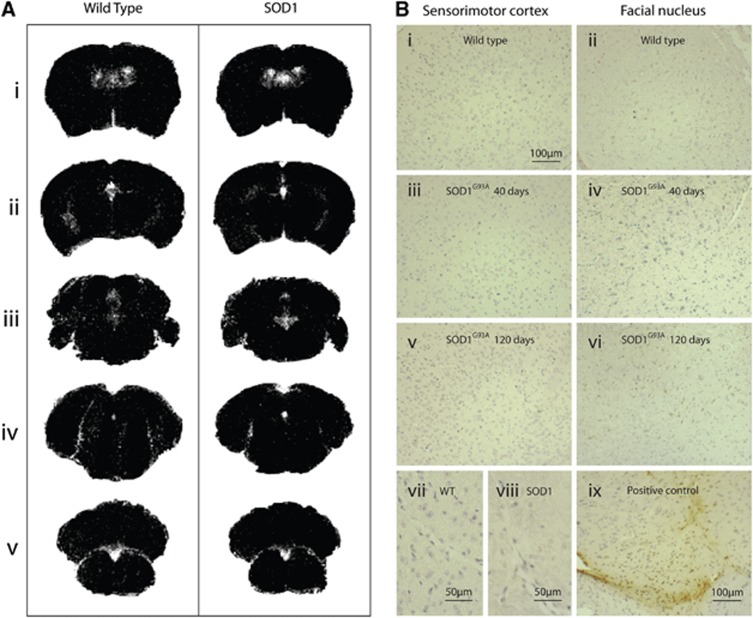
No evidence of blood–brain barrier (BBB) breakdown is seen at any time point in *SOD1*^*G93A*^ mice. Subtraction of Gadolinium *T*_1_ images (**A**) showed Gd-DTPA signal change in the ventricles in both *SOD1*^*G93A*^ and wild-type (WT) mice, but no signal change is seen in the tissue, either in the sensorimotor cortex (i and ii) or in any of the brainstem motor nuclei (iii–v). No IgG staining was noted at any time point (**B**) in WT (i and ii), 40-day *SOD1*^*G93A*^ (iii and iv), or 120-day *SOD1*^*G93A*^ (v and vi) tissue. Panels i, iii, and v show images from the sensorimotor cortex; ii, iv, and vi show images from nucleus VII. Similarly even at high magnification, no staining was seen around vessels in SOD1 (viii) or WT (vii) mice. Panel ix shows a photomicrograph from positive control tissue (experimental brain trauma) as a comparison. No change was seen in the spinal cord (images not shown). *N*=4 in each group. *SOD1*, superoxide dismutase; Gd-DTPA, gadolinium diethylene triamine pentaacetic acid.

*T*_1_ MRI using the contrast agent Gd-DTPA showed no increase in permeability in the vasculature of any region of the brain, at any time point of the disease (Figure 5A). This was confirmed by immunostaining for mouse IgG (Figure 5B). In contrast, significant mouse IgG immunoreactivity was found in brain trauma-positive control tissue (Figure 5Bix)

**Figure 6:**
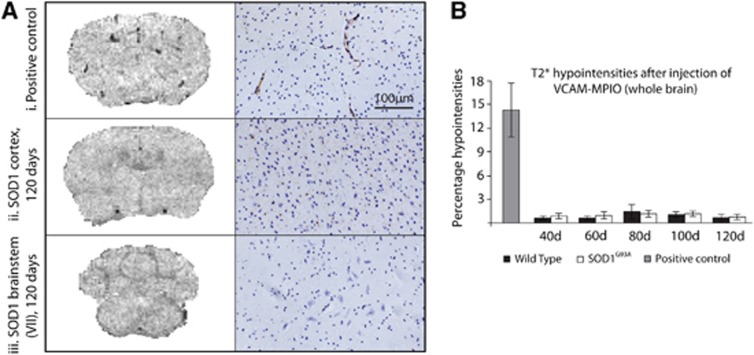
Imaging of VCAM-1 expression, and the corresponding immunohistochemistry, in the brains of *SOD1*^*G93A*^ mice and wild-type (WT) controls at 120 days, and positive interleukin-1*β* (IL-1*β*)-injected controls. Representative *T*_2_* images of the cortex (**A** ii, left panel) and brainstem (facial nucleus, **A** iii, left panel) in *SOD1*^*G93A*^ mice at end stage show no binding of VCAM-MPIO. However, significant binding could be seen after injection of IL-1*β* (**A** i, top left), where VCAM-MPIO binding causes signal dropout. This was confirmed by VCAM immunohistochemistry (**A**, right panels). Quantification of *T*_2_* hypointensities (**B**) shows that there was a significant VCAM-MPIO binding in the positive control only, with no significant binding in *SOD1*^*G93A*^ or WT mice at any time point. Data presented are for whole brain; analysis was also performed for the sensorimotor cortex and the brainstem in isolation, but no regional effects were found either (data not shown). Positive control *N*=3; *SOD1*^*G93A*^ and WT *N*=4. Scale bar applies to all immunohistochemistry images. VCAM-1, vascular cell adhesion molecule 1; MPIO, microparticles of iron oxide.

3D *T*_2_*-weighted data showed no evidence of VCAM-MPIO binding at any time point using a whole brain mask (Figure 6), nor was any increased binding seen when regional analysis was performed on the brainstem and sensorimotor cortex specifically (data not shown). Images shown are of the sensorimotor cortex (Figure 6Aii, left panel) and nucleus VII (Figure 6Aiii, left panel), but similar results were found in nuclei V and XII (data not shown). In contrast, IL-1*β*-injected mice (positive control) showed a high level of binding (Figure 6Ai, left panel, Figure 6B), especially in the region of the sensorimotor cortex. Immunohistochemistry for VCAM-1 was performed in *SOD1*^*G93A*^ mice and WT littermates and confirmed the very low level of VCAM-1 immunoreactivity in *SOD1*^*G93A*^ and WT mice (Figure 6A, right panels), showed very low immunoreactivity in the sensorimotor cortex, and cranial nuclei at any point from 40 to 120 days of age. There was also no significant VCAM immunostaining in WT or *SOD1*^*G93A*^ mice in the spinal cord at any time point (data not shown). However, in a model of experimental neuroinflammation using IL-1*β* injected into the striatum, there was significant VCAM-1 immunoreactivity at and around the site of injection, confirming the lack of VCAM-1 expression in *SOD1*^G93A^ mice.

## Discussion

This study showed the potential for MRI biomarkers of presymptomatic, in addition to symptomatic, disease in the *SOD1*^*G93A*^ mouse model of ALS, and explored the histopathologic basis for these changes. Our findings indicate that *T*_2_-weighted MRI is more sensitive to early pathologic changes than either ADC or MTR MRI, with *SOD1*^*G93A*^ pathology being detected in the facial nucleus as early as day 60. The MRI changes were accompanied by increased vacuolation, astrocyte activation, and microglia activation, and all three of these pathologic variables correlated to the same extent with the changes found with *T*_2_-weighted MRI. No changes in *T*_1_ signal intensity were observed at any time point, suggesting that the changes in *T*_2_ signal intensity and ADC were not due to simple accumulation of tissue fluid, but reflect more complex events. In contrast to previous reports, no change was found at any time point in either vascular permeability or endothelial activation, using both MRI and immunohistochemistry.

### *T*_2_-Weighted Magnetic Resonance Imaging Changes

This study highlights the potential advantage of using high field MRI in detecting the earliest pathologic changes in ALS. The progressive increase in *T*_2_ MRI signal observed in motor nuclei V (trigeminal), VII (facial), and XII (hypoglossal) in *SOD1*^*G93A*^ mice is consistent with previous studies that showed signal change either postsymptomatically or immediately before the onset of symptoms at 80 days.^7, 9, 10^ However, the present study found more dramatic changes in motor nucleus VII, beginning at 60 days, with a trend toward changes as early as 40 days. These changes were seen despite a relatively short scanning time (3 minutes 12 seconds) compared with that required for *T*_2_ mapping (77 minutes)^7^ or high-resolution *T*_2_ images (42 minutes).^9^ This increased sensitivity likely reflects, at least in part, the greater signal-to-noise achievable with higher field MRI systems, in this case 7 T compared with 4.7 T used previously.^7, 9^

The more pronounced signal change in nucleus VII is consistent with previous reports highlighting this region as a focus of degenerative changes, along with nucleus V.^9, 22^ However, one previous MRI study did not find any changes in the nucleus XII,^9^ and a histologic examination only found a trend toward loss of neurons in this nucleus.^22^ Results in the present study corroborate reports that nucleus XII does in fact show a robust pathologic change, at least in the *SOD1*^*G93A*^ model,^7^ although to a lesser extent than nuclei V and VII. The reduced effect of pathology in nucleus XII could be in part due to the nearby ventricle causing partial volume effects on MRI.

*T*_2_ MRI changes have been noted in human ALS. Standard *T*_2_ and fluid attenuated inversion recovery (FLAIR) sequences frequently show increased signal intensity in the corticospinal tract,^23^ but they lack sensitivity and specificity.^24^ However, standard clinical *T*_2_ sequences do not typically show pathology in the brainstem nuclei, even though pathology can be shown with other magnetic resonance applications such as spectroscopy,^25^ and through histopathologic analysis.^26^

### Histologic Correlates of Magnetic Resonance Imaging Changes

Both astrocytes and microglia underwent morphologic changes throughout the disease in *SOD1*^*G93A*^ mice. The earliest changes were found from 60 days for astrocytes and 80 days for microglia, and the protein expression continued to be elevated for the rest of the disease time course. Similarly, as shown by previous reports,^8^ early involvement of vacuole pathology was evident in all three cranial motor nuclei, in this case starting at 60 days and progressing in a fairly linear manner as the disease developed. Vacuole pathology, GFAP immunoreactivity, and Iba1 immunoreactivity were all significantly correlated with *T*_2_ signal change in nuclei V, VII, and XII, with no significant differences between any of the correlation coefficients in any of the regions. Previous reports have assumed that *T*_2_ signal change in cranial nuclei is due to the appearance of vacuoles, based solely on temporal coincidence.^8^ However, the data presented here show that any one of these parameters, (tissue vacuolation, astrocyte activation, and microglia activation), or some combination of the three might contribute to the MRI signal.

Since the nuclei in question are small structures (nucleus VII=∼0.85 mm diameter at widest point) it is not yet possible to draw any spatial correlations between the histopathologic features and MRI signal changes. While GFAP and Iba1 are constitutively expressed throughout the brain, the main areas of elevated GFAP expression in *SOD1*^*G93A*^ compared with WT are in the trigeminal, facial, and hypoglossal nuclei.

### Discrepancy between *T*_2_ and *T*_1_ Magnetic Resonance Imaging Signal Changes

As discussed above, previous studies have attributed *T*_2_ changes to the appearance of water filled vacuolation, which increases throughout the disease process.^8^ If these changes were a simple effect of increased water load on *T*_2_ relaxation, then a complementary change in *T*_1_ relaxation would also be expected.^27^ However, the data reported here show that this is not the case. Even at disease end point (120 days), when there is an ∼15-fold increase in *T*_2_ signal, there is no detectable change in *T*_1_ signal intensity. Thus, either the relationship between water load and *T*_2_ relaxation is more complex than previously suggested, or other pathologic features account for, or contribute to, the signal change. In support of the latter concept, we have previously showed a significant correlation between areas of *T*_2_ hyperintensity and astrocyte activation in a rat model of delayed type hypersensitivity central nervous system inflammation, in the absence of correlated changes in *T*_1_ signal intensity.^28^

### Magnetization Transfer Ratio and Diffusion-Weighted Imaging

Previous studies have shown a reduction in ADC values in both the brainstem and the spinal cord in *SOD1* compared with WT mice.^7, 29^ Consistent with these reports, at 120 days, a decrease in ADC values was observed in all three cranial motor nuclei. We additionally report a decrease in the MTR in nuclei V, VII, and XII. While both ADC and MTR values were significantly different between *SOD1* and WT groups, the effect size was less than for *T*_2_ signal change, suggesting that *T*_2_-weighted MRI would be the leading candidate biomarker of disease progression in *SOD1*^G93A^ mice. Our findings are also in line with human studies showing MTR changes in the posterior limb of the internal capsule and the precentral gyrus.^30, 31^ Similarly, changes in diffusion parameters specifically reduced fractional anisotropy and increased medial diffusivity in the rostral corticospinal tracts and central corpus callosum are a core feature of human ALS.^32^ *T*_2_ imaging, MTR, and diffusion changes have not been specifically reported in the brainstem nuclei in ALS patients, despite the pathologic involvement of the hypoglossal nuclei being common in relation to bulbar weakness in the human disease.

A reduction in ADC indicates restricted tissue diffusion, and has been suggested to reflect either intracellular oedema^33^ or hypercellularity owing to glial activation and inflammatory cell recruitment.^28^ Magnetization transfer ratio measures the transfer of magnetic resonance signal from protons in water that are associated with macromolecules to protons in free water. Reductions in MTR have, therefore, been linked with structural changes in tissue that changes the ratio of free and bound protons, such as demyelination,^34^ or generalized inflammatory processes.^18^ Thus, it seems likely that the changes in cell morphology owing to glial activation may significantly contribute to all three of these MRI measures (*T*_2_, ADC, and MTR). More in-depth experimental paradigms are needed to determine the influence of these factors individually on *T*_2_, ADC, and MTR signal changes. However, taking all of the MRI data together, it is hypothesized that a richer combination of factors affecting tissue diffusion properties underpin MRI changes, rather than a simple acquisition of excess fluid.

### Blood–Brain Barrier Integrity

Blood–brain barrier breakdown (mainly spinal cord),^11, 13^ vascular damage,^12^ and tight junction protein downregulation^13^ have been reported in molecular studies of *SOD1* mice. However, we found no evidence in any brain region, at any time point, of BBB compromise to the extent that would allow leakage of either Gd-DTPA or IgG into the parenchyma of the mouse brain.

There are reports of central nervous system Gd-DTPA leakage around the midbrain/interbrain region near the lateral ventricle in the *SOD1*^*G93A*^ rat model using MRI.^14, 15^ However, the WT images were not reported for comparison in that study and, as shown in the present study, signal increase around the lateral ventricles may otherwise be overinterpreted. Also, because the Gd-DTPA leakage in those studies was adjacent to the ventricle, there is the potential for ‘contamination' of the signal by the high signal intensity of the cerebrospinal fluid, and/or from Gd-DTPA in the cerebrospinal fluid due to a disrupted blood–cerebrospinal fluid barrier.

Other than simple species differences, we also considered whether a possible cause of the varying results was reduced exposure to pathogens. There is substantial evidence to suggest that peripheral inflammation can exacerbate central pathology,^35^ and certainly the mice in the present study were raised in individually ventilated cages with very low risk of systemic infection.

We conclude that, at least for *SOD1*^*G93A*^ mice, BBB breakdown does *not* appear to be a substantial aspect of pathology.

### Vascular Activation

In other neuroinflammatory diseases such as multiple sclerosis, endothelial activation has been shown to be more sensitive to BBB change than Gd enhancement.^36^ In the current study, however, using both a molecular MRI approach and an immunohistochemistry, no increases in VCAM-1 expression were detected at any time point in *SOD1*^*G93A*^ mice, in whole brain, sensorimotor cortex, or brainstem region. In contrast, previous studies have shown activation of other cellular adhesion molecules in *SOD1* models, such as intercellular adhesion molecule (ICAM-1) and melanoma cellular adhesion molecule (MelCAM).^11, 37^ While it is possible that the endothelial changes are limited to other cellular adhesion molecules and do not involve VCAM-1, this is contrary to our current conception of VCAM-1 as being an integral molecule in the process of adhesion and diapedesis of peripheral inflammatory cells.^38^ We conclude that there is currently no useful role for VCAM-MPIO as a biomarker in *SOD1* ALS.

## Conclusions

*T*_2_-weighted MRI is sensitive to presymptomatic pathologic change in *SOD1*^*G93A*^ mice, significantly earlier than previously reported, and is superior to ADC and MTR as a biomarker of *SOD1*^*G93A*^ pathology. Significant breakdown of the BBB was not a feature of this model, nor was there any evidence for upregulation of endothelial VCAM-1 expression. Magnetic resonance imaging data using *T*_1_, *T*_2_, ADC, and MTR support the hypothesis that complex changes to the diffusion properties of *SOD1*^*G93A*^ brainstem tissue underpin the MRI change, further supported by the fact that histologic measures of vacuolation, GFAP staining, and Iba1 staining are correlated to a similar extent with *T*_2_ intensity.

The histopathologic changes in human ALS and those of the *SOD1*^*G93A*^ mouse model differ in their focus. Significant evolutionary differences in the motor system with respect to the descending tracts,^39^ whereby the CST is significantly more developed in primates compared with rodents, are a major factor. Nevertheless, the consistent predilection of T_2_ hyperintensities for brainstem nuclei in the *SOD1*^*G93A*^ mouse offers important potential as a pharmacodynamic biomarker in preclinical therapeutic studies, particularly neuroprotective agents aimed at the presymptomatic phase, which is an area of increasing interest in the human disease.^40^
