# Supplementary figures and images for: T2-weighted MRI detects presymptomatic pathology in the SOD1 mouse model of ALS
Source: J Cereb Blood Flow Metab. 2014 Feb 5;34(5):785–93. doi: 10.1038/jcbfm.2014.19 (PMC4013759; doi:10.1038/jcbfm.2014.19)

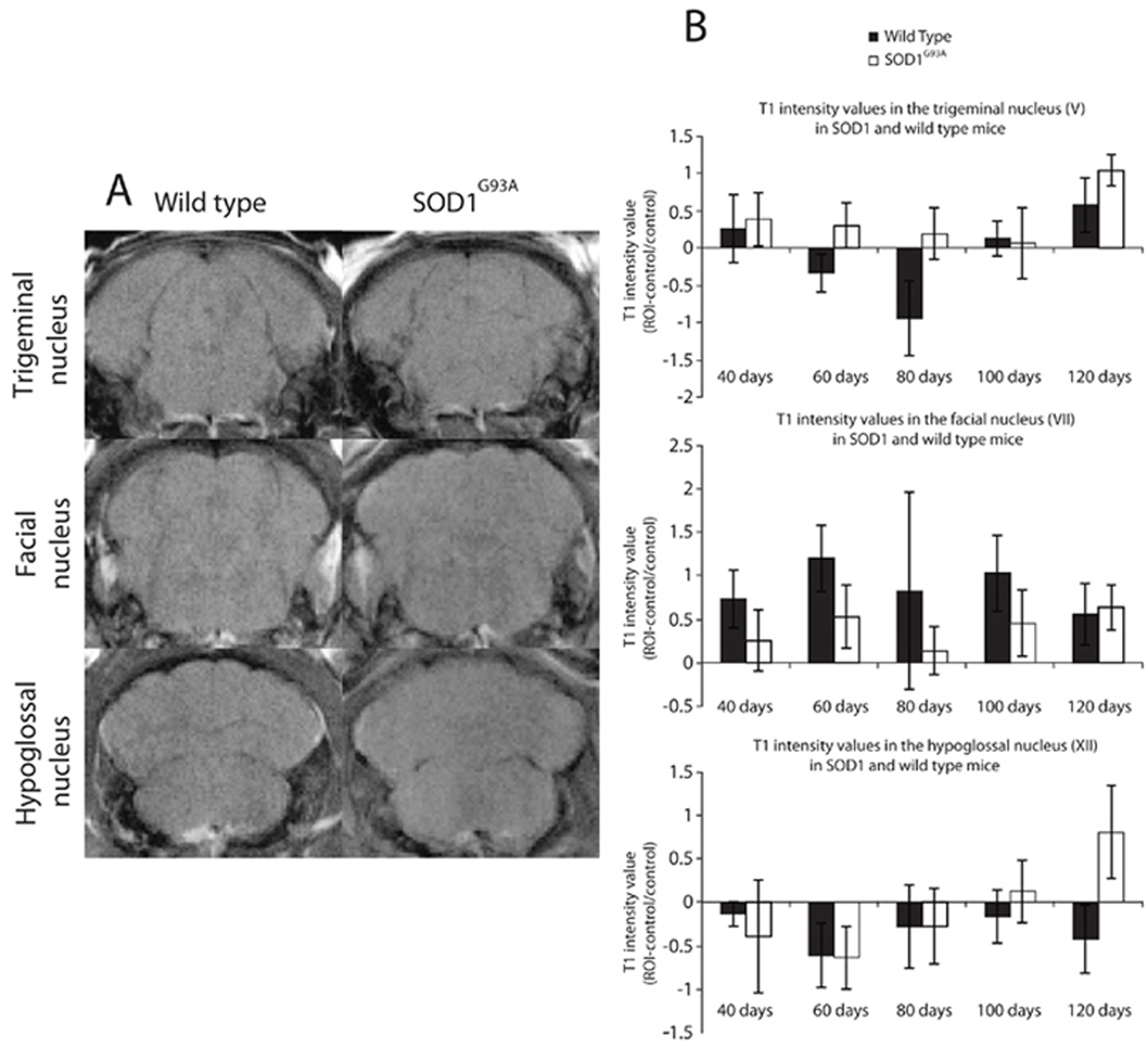

Supplement: Supplementary Figure 1 [file jcbfm201419x1.tif]

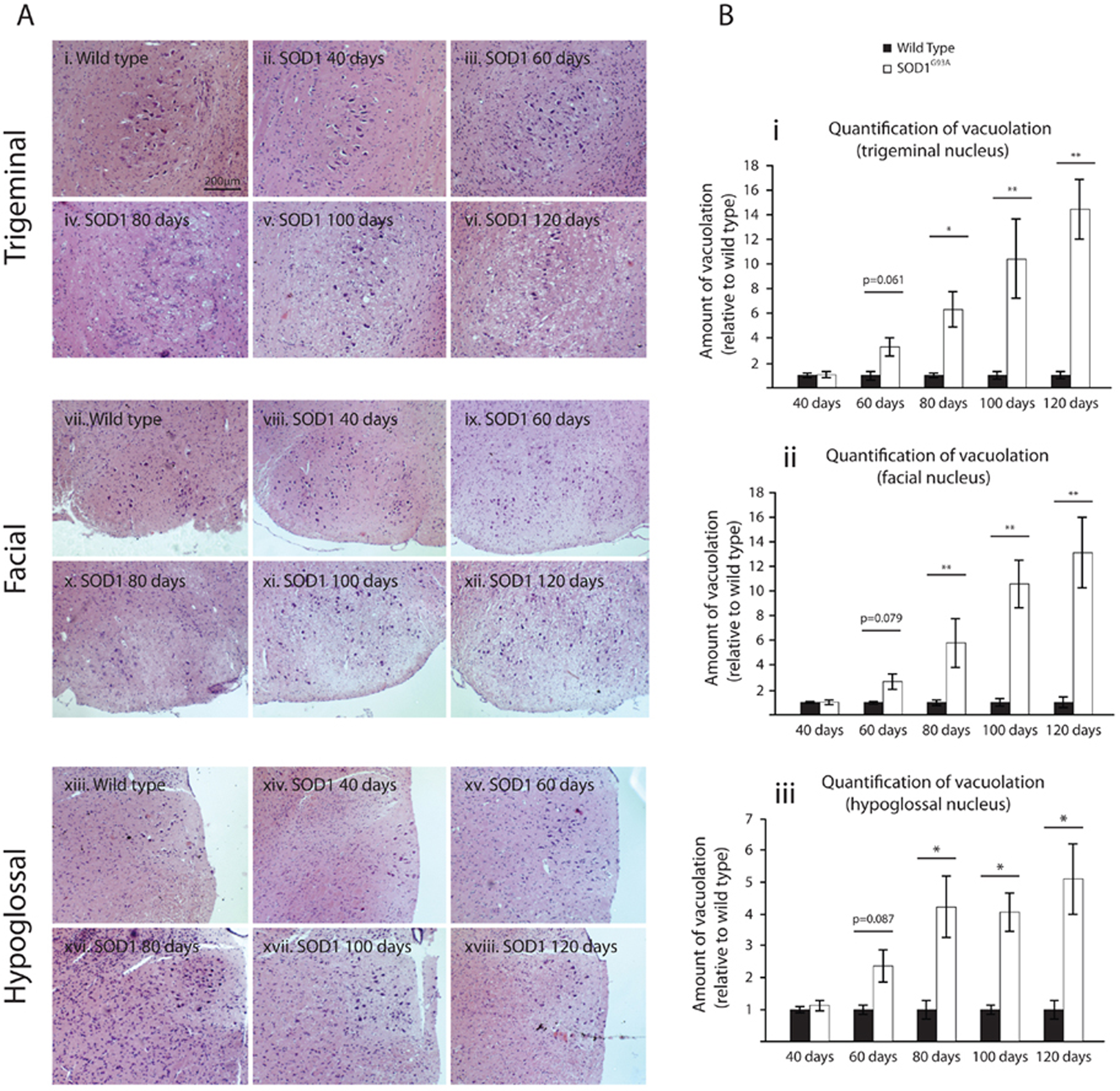

Supplement: Supplementary Figure 2 [file jcbfm201419x2.tif]

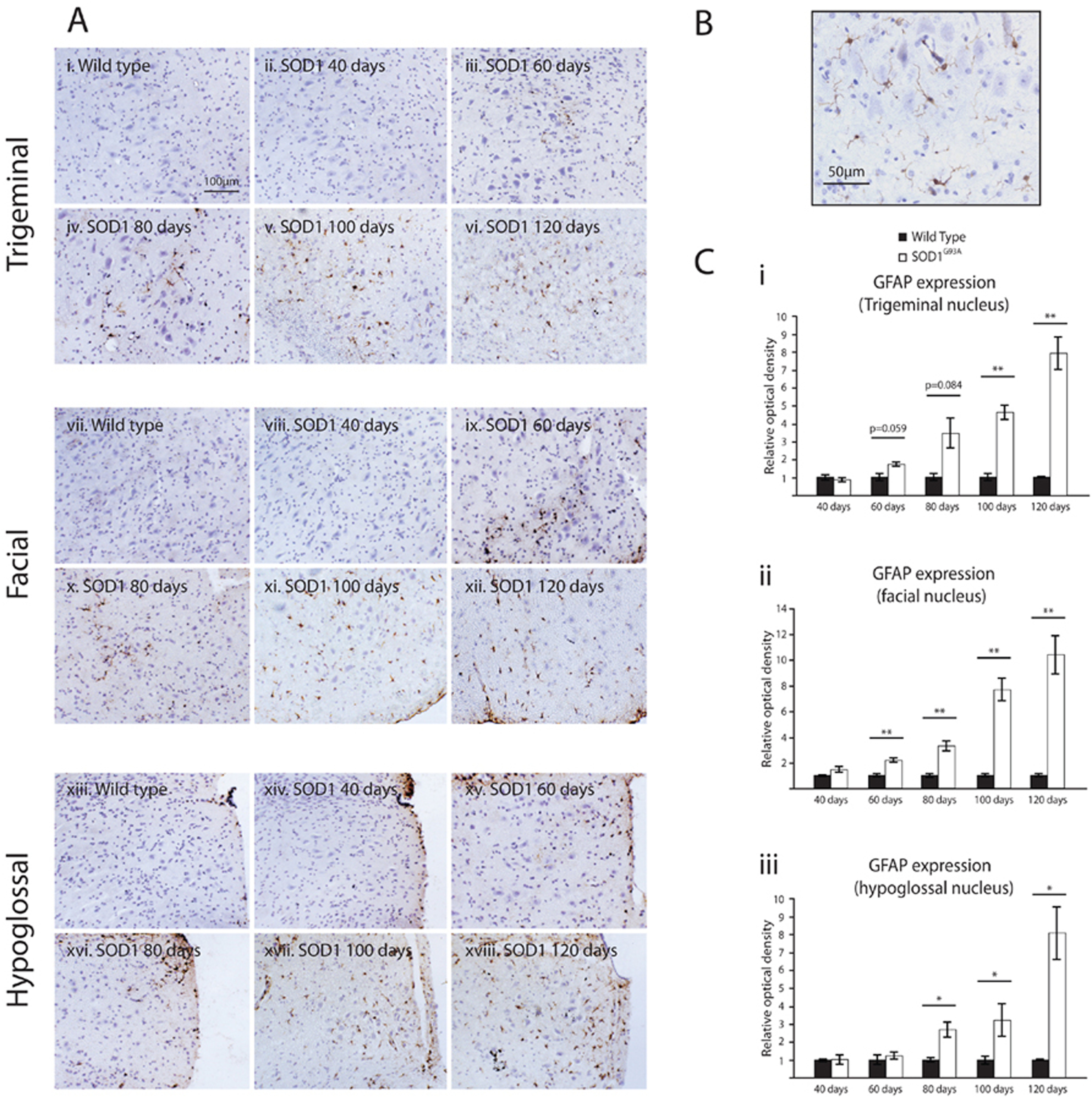

Supplement: Supplementary Figure 3 [file jcbfm201419x3.tif]

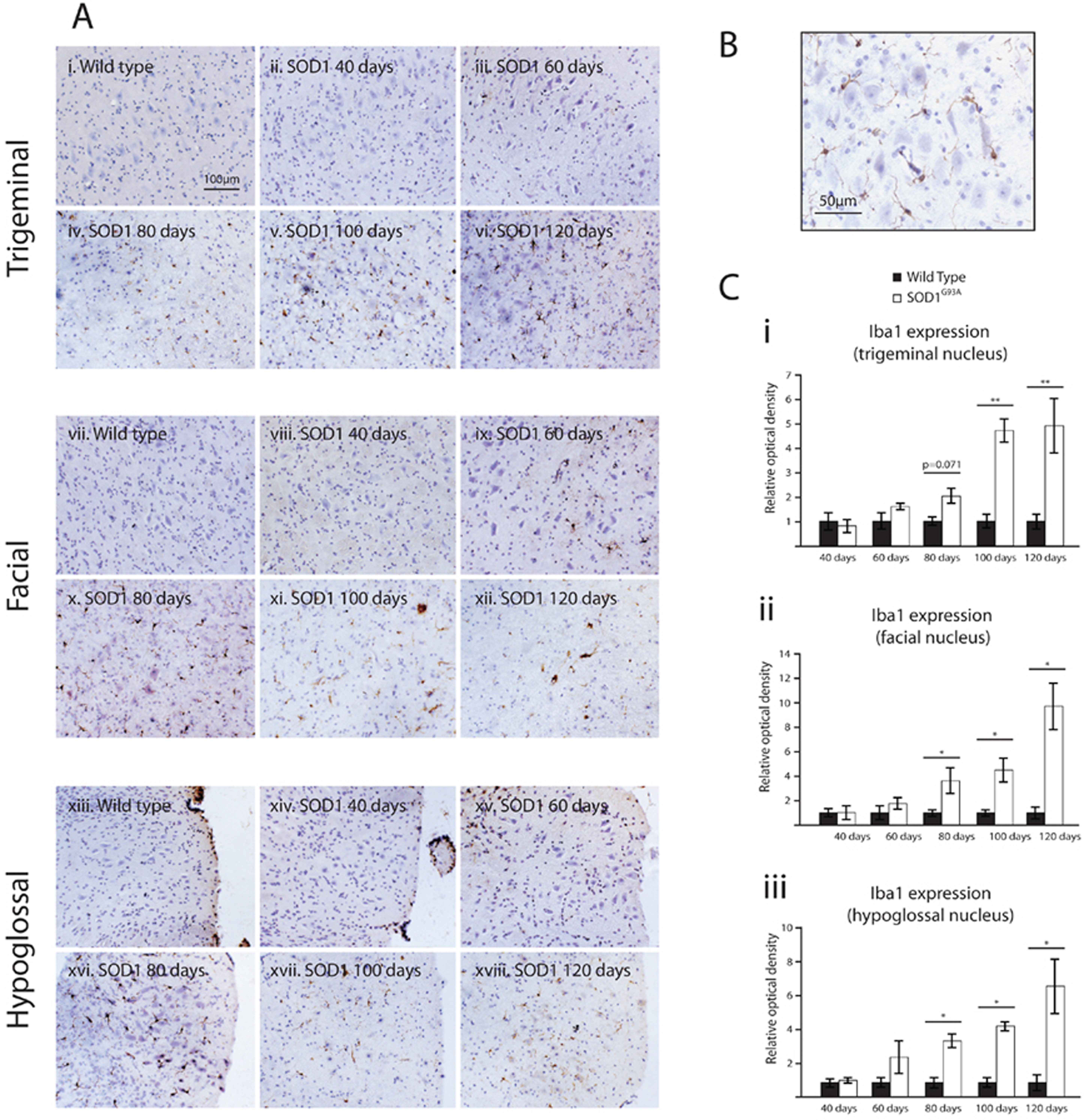

Supplement: Supplementary Figure 4 [file jcbfm201419x4.tif]
