# Supplementary material for: T2-weighted MRI detects presymptomatic pathology in the SOD1 mouse model of ALS
Source: J Cereb Blood Flow Metab. 2014 Feb 5;34(5):785–93. doi: 10.1038/jcbfm.2014.19 (PMC4013759; doi:10.1038/jcbfm.2014.19)
Supplement: Supplementary Figure Legends [file jcbfm201419x5.doc]

**Titles and Legends to Supplementary Figures**

**Supplementary Figure 1: Comparison of *T*_1_ intensity in the three motor nuclei in *SOD1^G93A^* and WT mice**

Representative *T*_1_ images of nuclei V, VII and XII (A) show homogenous *T*_1_ relaxation across the brainstem, with no noticeable changes in any of the nuclei in *SOD1^G93A^* or WT mice. Quantification of the *T*_1_ signal intensity in an ROI over each of the nuclei compared to a control region in the surrounding brainstem (B) shows no significant difference at any time point between genotypes.

N=4 in each group.

**Supplementary Figure 2: Development of vacuolation in the cranial motor nuclei in *SOD1^G93A^* compared with WT mice**

Representative H&E images of WT mice, and *SOD1^G93A^* mice throughout the disease process are shown in section A, for nuclei V (i-vi), VII (vii-xii) and XII (xiii-xviii). Vacuoles developed in the tissue from 60 days in the nucleus VII (ix-xii) and from 80 days in the V (iv-vi) and XII (xvi-xviii) in *SOD1^G93A^* mice. No vacuoles were found in these regions in WT littermates at any time point (i, vii, xiii). This pattern was confirmed by quantifying the amount of vacuolation in the tissue (section B).

* p<0.05; ** p<0.01; *** p<0.001. N = 4 in each group

**Supplementary Figure 3:** **GFAP immunohistochemistry in the cranial motor nuclei in *SOD1^G93A^* compared with WT mice**

Representative images of GFAP staining for wild type mice, and *SOD1^G93A^* throughout the disease process are shown in section A, for nuclei V (i-vi), VII (vii-xii) and XII (xiii-xviii). There was an increase in GFAP staining in nuclei V and VII at 60 days (iii-vi, ix-xii), and in the nucleus XII at 80 days (xvi-xviii) for *SOD1*^G93A^ mice, which became more prominent as the disease progressed. Immuno-reactivity to GFAP was very low in wild type mice (i, vii, xiii). This pattern was confirmed by quantifying the optical density for GFAP immuno-staining (section C). A high-resolution image of astrocytes showing typical morphology is shown in section B.

* p<0.05; ** p<0.01; *** p<0.001. N = 4 in each group

**Supplementary Figure 4: Iba1 immunohistochemistry in the cranial motor nuclei in *SOD1^G93A^* mice**

Representative images of Iba1 staining for wild type mice, and *SOD1^G93A^* throughout the disease process are shown in section A, for nuclei V (i-vi), VII (vii-xii) and XII (xiii-xviii). There was an increase in Iba1 staining in the all three cranial motor nuclei at 80 days in *SOD1*^G93A^ mice (iv-vi, x-xii, xvi-xviii), which became more prominent as the disease progressed. Immuno-reactivity to Iba1 was very low in wild type mice (i, vii, xiii). This pattern was confirmed by quantifying the optical density for Iba1 immuno-staining (section C). A high-resolution image of microglia showing typical morphology is shown in section B.

* p<0.05; ** p<0.01; *** p<0.001. N = 4 in each group
