# Supplementary material for: T2-weighted MRI detects presymptomatic pathology in the SOD1 mouse model of ALS
Source: J Cereb Blood Flow Metab. 2014 Feb 5;34(5):785–93. doi: 10.1038/jcbfm.2014.19 (PMC4013759; doi:10.1038/jcbfm.2014.19)
Supplement: Supplementary Tables [file jcbfm201419x6.doc]

**Tables (Supplementary)**

|  | **V** | **VII** | **XII** |
| --- | --- | --- | --- |
| **40** | -1.22 | 2.32 | 0.32 |
| **60** | 0.94 | 11.34 | 1.41 |
| **80** | 8.22 | 18.89 | 7.55 |
| **100** | 10.14 | 16.35 | 6.56 |
| **120** | 8.58 | 17.65 | 14.86 |

**Supplementary Table 1: Effect size (Cohen’s d) for** *T*_2_ **intensity in *SOD1^G93A^* compared to WT mice from 40 to 120 days**

Effect size for the *T*_2_ intensity in *SOD1^G93A^* compared to WT mice is markedly higher in the nucleus VII compared to nuclei V or XII at all time points. Effect sizes in nuclei V and XII are mostly comparable.

|  | **T2** | **MTR** | **ADC** |
| --- | --- | --- | --- |
| V | 12.04 | 6.33 | 9.58 |
| VII | 27.98 | 7.39 | 7.19 |
| XII | 9.01 | 8.72 | 5.72 |

**Supplementary Table 2 – Effect size (Cohen’s d) for *T*_2_, MTR and ADC sequences at 120 days in brainstem motor nuclei, for *SOD1^G93A^* compared with WT mice**

Effect size for the difference between *SOD1^G93A^* and WT mice was highest in all three nuclei for the *T*_2_ sequence. Effect size in nucleus VII was comparable for MTR and ADC, whereas there was a higher effect size associated with ADC in nucleus V, and a higher effect size for MTR in nucleus XII.
